# Rosmarinic Acid and* Melissa officinalis* Extracts Differently Affect Glioblastoma Cells

**DOI:** 10.1155/2016/1564257

**Published:** 2016-09-05

**Authors:** Kristina Ramanauskiene, Raimondas Raudonis, Daiva Majiene

**Affiliations:** ^1^Department of Clinical Pharmacy, Lithuanian University of Health Sciences, Sukileliu st. 13, LT-50166 Kaunas, Lithuania; ^2^Department of Pharmacognosy, Lithuanian University of Health Sciences, Sukileliu st. 13, LT-50166 Kaunas, Lithuania; ^3^Laboratory of Biochemistry, Neuroscience Institute, Lithuanian University of Health Sciences, Eiveniu str. 4, LT-50009 Kaunas, Lithuania

## Abstract

Lemon balm (*Melissa officinalis *L.) has many biological effects but especially important is its neuroprotective activity. The aim of the study is to produce different extracts of* Melissa officinalis *and analyse their chemical composition and biological properties on rat glioblastoma C6 cells. Results revealed that rosmarinic acid (RA) is the predominant compound of lemon balm extracts. RA has cytotoxic effect on glioblastoma cells (LC_50_ 290.5 *μ*M after the incubation of 24 h and LC_50_ 171.3 *μ*M after 48 h). RA at concentration 80–130 *μ*M suppresses the cell proliferation and has an antioxidant effect. 200 *μ*M and higher concentrations of RA have a prooxidant effect and initiate cell death through necrosis. The aqueous extract of lemon balm is also enriched in phenolic compounds: protocatechuic, caftaric, caffeic, ferulic, and cichoric acids and flavonoid luteolin-7-glucoside. This extract at concentrations 50 *μ*M–200 *μ*M RA has cytotoxic activity and initiates cell death through apoptosis. Extracts prepared with 70% ethanol contain the biggest amount of active compounds. These extracts have the highest cytotoxic activity on glioblastoma cells. They initiate generation of intracellular ROS and cell death through apoptosis and necrosis. Our data suggest that differently prepared lemon balm extracts differently affect glioblastoma cells and can be used as neuroprotective agents in several therapeutic strategies.

## 1. Introduction

Glioblastoma multiforme (GBM), the most common and the most lethal CNS cancer, causes approximately 50% of all brain tumors. Chemotherapy and radiotherapy are used for treatment. Temozolomide is a first-line medicament for treatment of GBM; however this drug has many adverse effects and average lifespan of patients after treatment is about 1 year. Therefore, nowadays the fact that a “single” targeted therapy might not be the most effective approach and multitargeting would be the rational approach for killing a heterogeneous population of cancer cells in a tumor is often discussed. The relevance of natural agents of dietary origin in human cancer is appreciated, because it is a part of the normal diets in various cultures, these agents are nontoxic to humans and are able to modulate multiple signalling pathways [1]. It is important to determine if natural bioactive compounds (extracts of* Melissa officinalis* and rosmarinic acid), the neuroprotective effect of which is proven, could be potential candidates for additional therapy in brain cancer cases.

Lemon balm (*Melissa officinalis*) and its main active substance, rosmarinic acid (RA), have multiple neuroprotective effects. Scientific data show that rosmarinic acid could decrease level of intracellular reactive species and the level of DNA damage induced by ethanol in mice [2]. RA produces a significant neuroprotective potential in rats with ischemia and reperfusion: it reduces apoptosis and necrosis, increases cell survival, and decreases LDH leakage rate in cultured SH-SY5Y cells [3]. Pre- and posttreatment with RA decrease ciguatoxin-mediated neurotoxicity diminishes the extracellular LDH activity and DNA damage in primary human neurons [4]. RA exhibits neuroprotective effects in the neurotoxicity of amyloid *β*- (A*β*-) induced cognitive dysfunction and has an antidepressant-like property in animal models of depression [5]. It is now widely studied RA anticancer activity. RA was applied to various human cancer cell lines like NCI-H82, DU-145, Hep-3B, K-562, MCF-7, PC-3, MDA-MB-231, and it was shown that RA may inhibit cell proliferation, induce apoptosis, and decrease viability of investigated cells in dose-dependent manner [6, 7] Since the number of brain cancer cases has increased in the past decades and the effects of RA on tumor cells are not clearly identified, the aim of this work is to identify the effects of this substance on the most aggressive type of brain tumors, glioblastoma cells.

A lot of research is being performed in order to prove the effect of extracts, made from* Melissa officinalis,* in treatment of different forms of brain diseases [8–14]. Investigations on rats have been performed, which established that the aqueous or methanolic extract of lemon balm affects the GABA transaminase in the brain as an inhibitor in anxiety, epilepsy, and so forth, [8, 9]. According to Akhondzadeh et al. the effect is achieved by stimulating the activity of acetylcholine receptors in the central nervous system [10]. Scientific data has proven that* Melissa officinalis* extracts may have anticancer activity. Encalada with coauthors demonstrated cytotoxic effect of the 50% ethanolic and aqueous extract against human colon cancer cells [15]. Weidner with coauthors evaluated the effect of ethanolic lemon balm extract on HT-29 and T84 human colon carcinoma cells. Experimental data showed that investigated extract inhibits the proliferation of colon carcinoma cells and induces apoptosis through formation of ROS [16].

Most studies have shown that the biological effect of* Melissa officinalis* extracts, as well as other plants from the* Lamiaceae* family, mainly depends on RA concentration [11–13]. Differently prepared extracts are enriched with other biologically active compounds, which, acting synergistically with RA, may increase biological effect of the extracts. Extracts are produced by using various extraction solvents. Water as solvent is always used in preparation of extract (tea) at home. However, most of biologically active compounds have low solubility in water. Ethanol is the most popular solvent in industry for producing liquid extracts intended for oral use. Additionally, ethanol is capable of dissolving most of biologically active compounds nonsoluble in water. In summary, the amount of active compounds in differently prepared extracts depends on solvent and production conditions.

The aim of this study was to produce different liquid extracts of lemon balm (*Melissa officinalis* L.) and analyse the chemical composition, investigate their antiproliferative, antioxidant, and cytotoxic effects on rat glioblastoma C6 cells, and compare them with effects of rosmarinic acid.

## 2. Materials and Methods

### 2.1. Chemicals and Reagents

Raw lemon balm (*Melissae folium*) was obtained from JSC “*Acorus calamus*” (Vilnius, Lithuania), and dry lemon balm extract (*sicc. Extractum Melissae officinalis*) was obtained from Naturex (France). All the reagents and standards were of analytical grade. Luteolin-7-glucoside, caffeic acid, and rosmarinic acid were obtained from Extrasynthese (Genay, France), protocatechuic acid, caftaric acid, ferulic acid, and cichoric acid from Fluka (Buchs, Switzerland). Dulbecco's modified Eagle's medium (DMEM), Ampliflu*™* Red, and 2′,7′-dichlorodihydrofluorescein diacetate (DCFH_2_-DA), 3-(4,5-dimethylthiazol-2-yl)-2,5-diphenyl-tetrazolium bromide (MTT), HPLC-grade acetonitrile, and trifluoroacetic acid (TFA) obtained from Sigma-Aldrich GmbH (Buchs, Switzerland). Deionized water was acquired from a Milli-Q purification system (Bedford, USA).

### 2.2. Preparation of Lemon Balm Extracts

The lemon balm dry extract was dissolved in purified water at the ratio 1 : 100. Manufactured extract (N1) is filtered through paper filter.

Extracts of lemon balm (N2, N3) are produced using 40% and 70% ethanol solutions as extract solvents. Raw material and extract solvent ratio is 1 : 1. Crushed herbal of lemon balm is soaked in an appropriate amount of solvent and left for maceration for seven days [14]. After extraction, extracts of lemon balm were filtered through paper filter.

### 2.3. Analysis of Extracts by High-Performance Liquid Chromatography

Chromatographic analysis was carried out using Waters Alliance e2695 Separations Module equipped with a Waters 2998 PDA Detector (Milford, USA). The separation was performed on an ACE Excel 3 SuperC18 analytical column (Aberdeen, Scotland) (250 × 4.6 mm, 3 *μ*m) at 25°C. The mobile phase consisted of 0.1% TFA in deionized water (A) and acetonitrile (B). The gradient elution was as follows: 0–30 min, 15%–30% B; 30–50 min, 30%–60% B; 50–55 min, 60%–90% B; and 55–60 min, 90%–15% B. The flow rate was 0.5 mL min^−1^, and the injection volume was 10 *μ*L. The detector was set in the 200–400 nm range. The chromatographic data were acquired and processed with Empower 3 software (Milford, USA).

### 2.4. Cell Line and Cell Culture

The C6 rat glioma model has been widely used in experimental neuro-oncology to evaluate the therapeutic efficacy of a variety of modalities, including chemotherapy [17]. A unique feature of C6 culture is that, exactly like a GBM tumor, it contains a subculture of cancer stem cells that express CD133 and nestin, which are widely used markers for brain CSCs [18]; therefore we choose this cell line for our investigations.

Rat glioblastoma C6 cells were purchased from the Cell Lines Service GmbH (Germany). C6 cells of convenient concentration were seeded in culture flasks containing DMEM with 10% of fetal bovine serum, 100 U/mL penicillin and 100 *μ*g/mL streptomycin. The cultures were then incubated at 37°C, with 5% CO_2_ and saturated humidity; culture transfer was performed once every 3-4 days.

### 2.5. Assessment of Cell Viability

#### 2.5.1. MTT Assay

Cell viability was assessed by measuring the ability of cells to metabolize MTT. After incubation of C6 cells in 96-well plates (20000 cells/well) for 24 h, they were treated without (control) or with different concentrations of investigated solutions for 24 or 48 h. After treatment DMEM medium was removed from wells, and then cells were washed twice with 100 mL/well Phosphate Buffered Saline (PBS). After washing, 180 *μ*L/well PBS was added along with 20 *μ*L/well of 5 mg/mL MTT dye dissolved in PBS to each well. The cells were incubated with MTT for 2 h. Blue Formosan crystals formed in the intact cells were dissolved in DMSO (100 *μ*L/well). The absorption was measured at 570 nm and 620 nm as reference with a microplate spectrophotometer (Sunrise, Tecan Group Ltd., Switzerland). The results were expressed as percentages of MTT reduction, with the absorbance exhibited by the control cells being as 100%.

Concentration of ethanol used for control varied depending on the amount of extracts used for investigation, the maximal concentration was 2.5% of ethanol.

The LC_50_ concentration was calculated by nonlinear regression analysis, fitting the data to equations, using the software package SigmaPlot 12.0 version (Systat Software Inc.).

#### 2.5.2. Assessment of Cell Count and Cell Death by Hoechst and Propidium Iodide Staining

Cell count was assessed using Hoechst 33258 and propidium iodide staining. At first, C6 cell suspension was dispersed to 24-well plates (2500 cells/well). After 24 h different concentrations of analysed preparations were added to the medium for 24 h, 48 h, and 72 h. After treatment, 15 min before investigation 5 *μ*g/mL of Hoechst 33258 and 2 *μ*g/mL of propidium iodide were directly added to the culture medium. After incubation viable, apoptotic, and necrotic cells were counted under fluorescence microscope.

### 2.6. Measurement of Intracellular RS Generation

The production of RS was assessed using the 2′,7′-dichlorofluorescein diacetate (DCFH-DA). After incubation of C6 cells in 96-well plates (20000 cells/well) for 24 h, they were incubated with DCFH-DA (10 *μ*M) in HBSS at 37°C for 30 min. During this time a part of DCFH is diffused into the cells. The dye, which was not diffused into the cells during incubation and remained in the outside medium, was washed twice with PBS. Wells were filled with a HBSS medium, enriched with investigated solutions of different concentrations. In the presence of cellular oxidizing agents, DCFH is oxidized to the highly fluorescent compound dichlorofluorescein (DCF); thus, the fluorescence intensity is proportional to the amount of RS produced in the cells. The fluorescence of DCF was detected by fluorometer at excitation and emission wavelengths of 488 and 525 nm, respectively.

### 2.7. Statistical Analysis

Results are presented as means ± standard error. Statistical analysis was by one-way analysis of variance (ANOVA), followed by Dunnett's posttest using the software package SigmaPlot 12.0 version (Systat Software Inc.). A value of *p* < 0.05 was taken as the level of significance.

## 3. Results

### 3.1. Chemical Composition of Differently Prepared Extracts

The chemical composition of the lemon balm extracts as obtained by HPLC method is shown in Table 1. Results revealed that RA is the predominant active ingredient of lemon balm extracts. Since RA is soluble in water, its concentration in aqueous extracts is relatively high, although the concentration of other identified substances is higher in ethanol solutions. Extracts prepared with 40% ethanol contain significantly smaller amount of all identified compounds in comparison with 70% ethanol. Results of our study demonstrated that 70% ethanol is the best solvent for extracting biologically active compounds from lemon balm.

### 3.2. Effect of Analysed Preparations on Cell Viability

**Figure 1:**
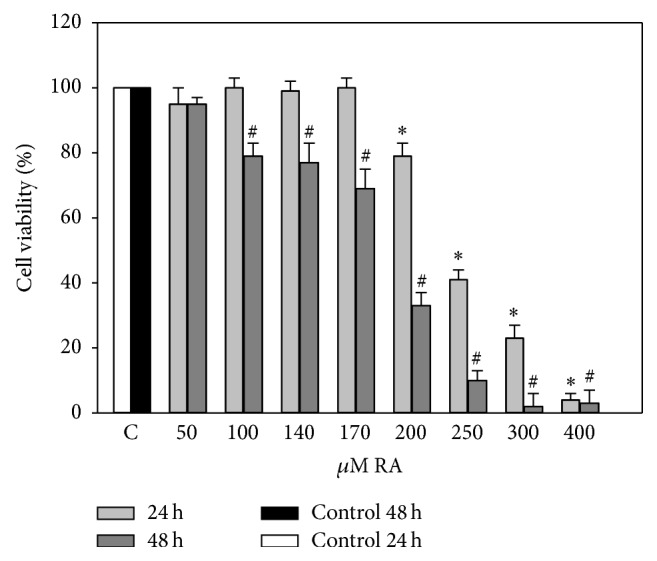
Effects of different concentrations of RA on the viability of C6 cells. C6 cells were treated with different concentrations (50–400 *μ*M) of RA for 24 and 48 hours. Cell viability was assessed using MTT method. Data are presented as means of percentage of the untreated control cells ± SE (*n* = 5). ^*∗*^ *p* < 0.05 versus control 24 h, ^#^ *p* < 0.05 versus control 48 h.

Investigation of biological activities was started from analysis of the effect of various RA concentrations on viability of C6 cells. As estimated by MTT assay (Figure 1), cell viability was significantly decreased only at a concentration of 200 *μ*M and higher after 24 h incubation. Therefore we extended the incubation with various RA concentrations up to 48 h. After 2 days a statistically significant decrease in viability was determined at 100 *μ*M RA concentration. Calculated RA LC_50_ after 24 h of incubation was 290.5 ± 48.1 *μ*M, and after 48 h LC_50_ was 181.3 ± 20.4 *μ*M.

**Figure 2:**
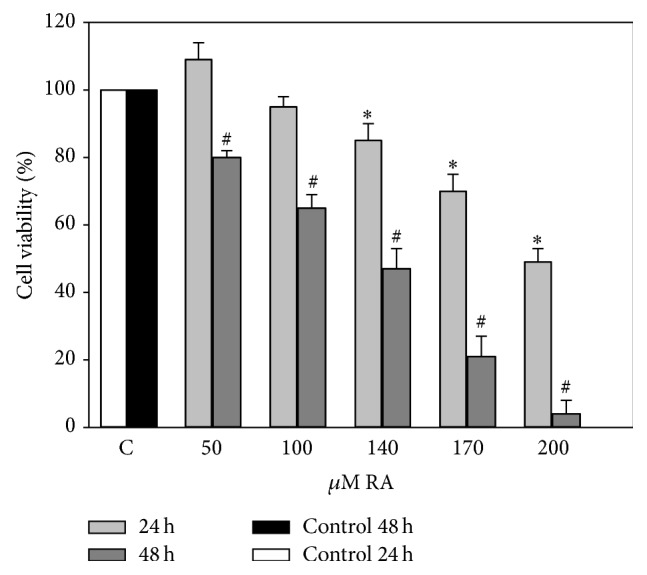
Effects of different concentrations of aqueous extract N1 on the viability of C6 cells. C6 cells were treated with different concentrations of aqueous extract (50–200 *μ*M of RA) for 24 and 48 hours. Cell viability was assessed using MTT method. Data are presented as means of percentage of the untreated control cells ± SE (*n* = 4-5). ^*∗*^ *p* < 0.05 versus control 24 h, ^#^ *p* < 0.05 versus control 48 h.

Extract N1 (Figure 2), produced by using a water solvent, after 24 h of incubation at a lowest investigated amount (RA concentration 50 *μ*M), had tendency to increase intensity of absorption. A statistically significant decrease of viability was determined in an amount of extract with an RA concentration of 140 *μ*M or higher. After extending the period of incubation to 48 h, a decrease in viability was estimated at 50 *μ*M RA, and a 100% cell death was caused by amount of extract N1, containing 200 *μ*M RA.

**Figure 3:**
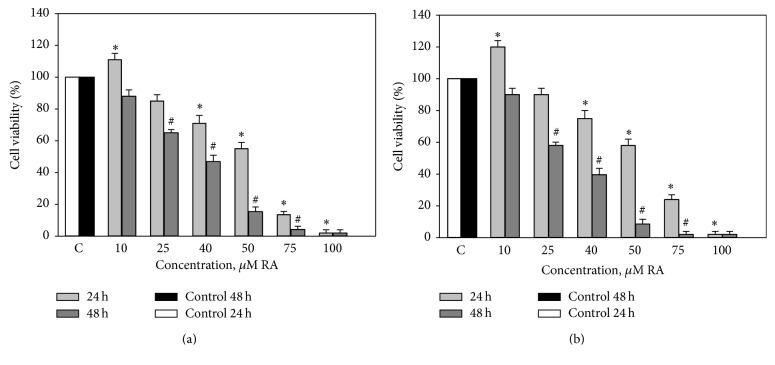
Effects of different concentrations of ethanolic extracts (a) N2 and (b) N3 on the viability of C6 cells. C6 cells were treated with different concentrations of ethanolic extracts (10–100 *μ*M of RA) for 24 and 48 hours. Cell viability was assessed using MTT method. Data are presented as means of percentage of the untreated control cells ± SE (*n* = 3). ^*∗*^ *p* < 0.05 versus control 24 h, ^#^ *p* < 0.05 versus control 48 h.

40% and 70% ethanolic extracts have affected cell viability significantly stronger (Figures 3(a) and 3(b)) if compared with N1 or RA alone. In both cases when the amount of investigated extracts was small (10 *μ*M of RA), cell viability has increased after 24 h of incubation (11–19%), extracts with 40–100 *μ*M RA have statistically significantly decreased cell viability by 25–98% after 24 h of incubation. After 48 h of incubation the viability decreased by 50% with extracts containing 40 *μ*M RA and more.

### 3.3. Assessment of Antiproliferative and Cytotoxic Effect of Analysed Preparations

**Figure 4:**
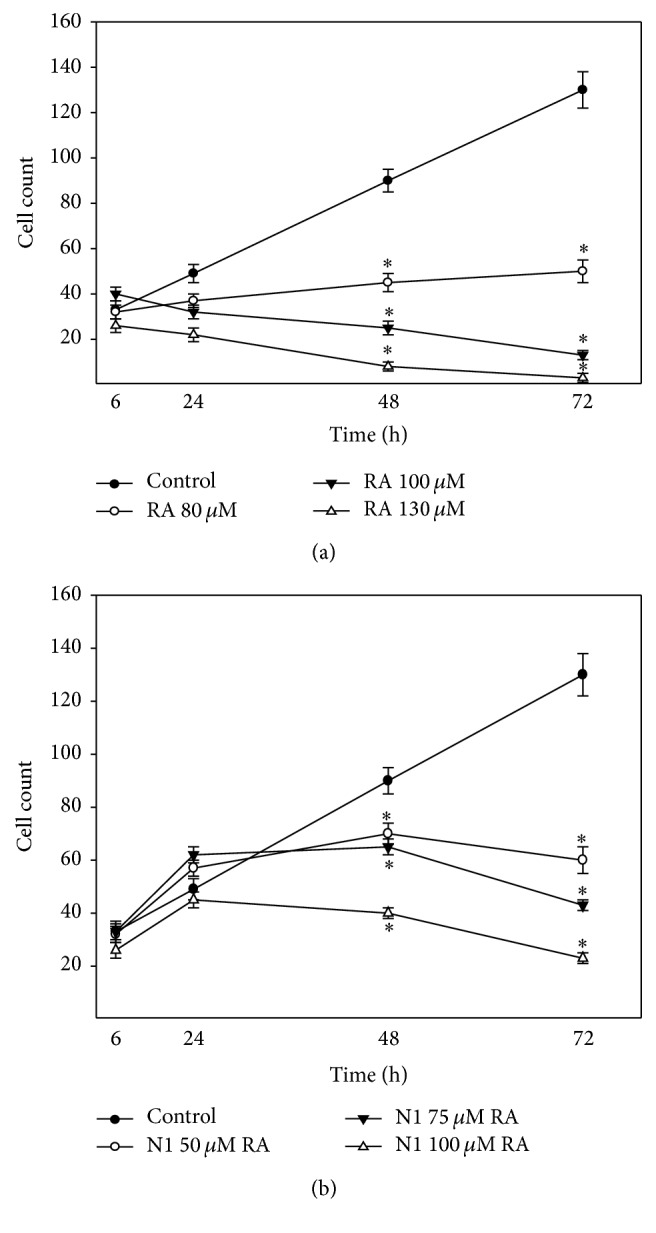
Effect of different concentrations of (a) RA and (b) N1 on C6 cell proliferation. Cell count was performed by adding Hoechst 33258 and propidium iodide dyes and counting the cells under fluorescence microscope. Data are presented as means of cell counts ± SE (*n* = 4-5). ^*∗*^ *p* < 0.05 versus control.

**Figure 5:**
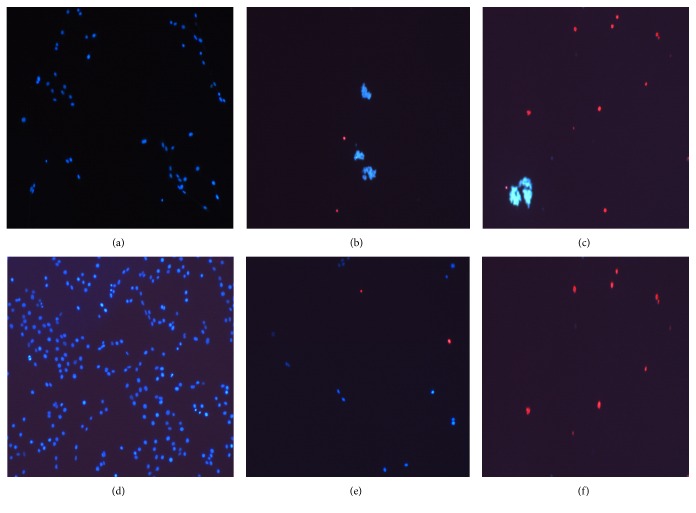
Typical images of glioblastoma C6 cells after incubation with different concentrations of RA and extracts. (a) Control after 24 h incubation; (b) N1 200 *μ*M RA; (c) N3 50 *μ*M RA; (d) control after 48 h incubation; (e) 170 *μ*M RA; (f) 200 *μ*M RA.

Glioblastoma cells have a very high proliferative activity; therefore it is relevant to analyse proliferation-inhibiting properties of investigated preparations. RA concentrations not yet causing cell death (80–130 *μ*M) have been chosen for this analysis. Results (Figure 4(a)) have shown that after 6 h the number of cells did not differentiate in any group. After 24 h the number of cells treated with 130 *μ*M RA has decreased in comparison with the control. Significant changes have been evident after 48 h and 72 h of incubation: the cells in the control group gradually multiplied, whereas the cell count in groups incubated with RA (80 *μ*M) has unchanged or started to decrease (100 *μ*M, 130 *μ*M) and some necrotic cells were found. Higher RA concentrations cause cell death by necrosis (Figures 5(d), 5(e), and 5(f)).

Different results were found by analysing the antiproliferative and cytotoxic activity of N 1–3 extracts.

The aqueous extract N1, used in concentrations 50 *μ*M–100 *μ*M RA (Figure 4(b)), has increased or has not changed the number of cells after 24 h incubation and decreased the number after 48 and 72 h. This extract caused death of C6 cells mainly by apoptosis (89 ± 4%) and only a small number of cells died due to necrosis (12 ± 3%) (Figures 5(a) and 5(b)).

The number of cells has increased in extracts N2 and N3 with 5–10 *μ*M RA concentration, whereas higher concentrations resulted in dead cells. Cell death by apoptosis (61 ± 7%) and necrosis (34 ± 5%) has been determined (Figure 5(c)).

### 3.4. Assessment of Antioxidant/Prooxidant Activity of Analysed Preparations

Since the extracts in low concentrations increased the proliferation of cells we have hypothesised that this effect can be initiated by an increased amount of intracellular reactive species (RS) and our further experiments were aimed to assess effect of the analysed preparations on the concentration of RS.

**Figure 6:**
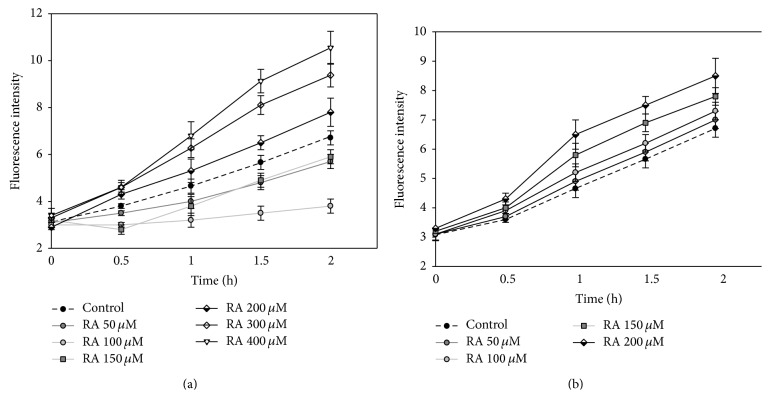
The effect of (a) RA and (b) N1 extract on concentration of intracellular RS. C6 cells were pretreated with 10 *μ*M DCFH-DA and then treated with different concentrations of RA and aqueous extract N1 for 0; 0.5; 1; 1.5; and 2 hours. Fluorescence intensity, which is proportional to intracellular RS concentration, was detected by using a fluorometer at excitation and emission wavelengths of 488 and 525 nm, respectively. Data is presented by fluorescence intensity ± SE (*n* = 3).

At first we analysed the effect of RA on the amount of RS. The results show (Figure 6(a)), that RA at concentration of 50–150 *μ*M after 2 h reduced the amount of RS by 15–43%. 200 *μ*M and higher concentration of RA increased the amount of intracellular RS (16%, 40%, and 57% at, resp., 200 *μ*M, 300 *μ*M, and 400 *μ*M RA concentrations).

All concentrations of aqueous extract increased the amount of RS (Figure 6(b)); a statistically significant difference was achieved at concentrations of 150 *μ*M and 200 *μ*M, which after incubation of 2 h increased the amount of RS by 16% and 27%.

All investigated concentrations of N2 and N3 extracts (Table 2) increased the amount of intracellular RS and after 2 h it was ×1.86–2.23 higher at RA concentration of 75 *μ*M.

## 4. Discussion

Studies have suggested that diets rich in RA and RA rich extracts together with other phenolic compounds may exert neuroprotective effects in neuroinflammation, and neurodegeneration, as well as chemical-induced neurotoxicity and oxidative stress. Earlier studies revealed that preparations from* Melissa officinalis* may act as a modulator of mood and cognitive function and has antidepressant effect [19–22]. There is an increasing interest to identify plant-derived natural products with antitumor activities [23]; therefore we seek to investigate the effect of RA alone and its rich extracts, made of lemon balm, on glioblastoma cells.

Our experiments have shown that RA alone, depending on its concentration, has a proliferation-inhibiting effect. Results showed that, after an incubation of 48 and 72 hours, RA at concentrations 80–130 *μ*M efficiently inhibited the cell proliferation. Makino with coworkers demonstrated that DNA synthesis, stimulated by PDGE and TNF-*α* was significantly decreased by RA (IC_50_ 1.4 *μ*g/mL and 3.8 *μ*g/mL) and in process of cell proliferation RA might regulate DNA synthesis both in early and in late signal transduction [24]. However, there are experiments demonstrating that RA showed proliferative effects rather than cytotoxic activity in almost all cell lines tested with the highest effect in K-562 cells, exhibiting a cell viability of 205% at 139 *μ*M [6]. Our experiments revealed that RA reduces C6 cell viability; however, rather very high RA concentrations are needed to achieve this effect (LC_50_ after 24 h and 48 h incubation 290.51 *μ*M and 171.3 *μ*M, resp.) and only necrotic cells were found (Figures 5(e) and 5(f)). However, Hur with his coworkers found that after 24 h treatment of RA (3–30 *μ*M) Jurkat cells displayed apoptosis, and at least 48 h of incubation with RA was required to induce apoptosis in almost 80–100% of the cells [25]. Moon and coworkers reported that RA alone exhibited little effect on the cell viability in human leukemia cells. However, a combination of TNF-*α* and RA induced apoptosis [26]. Even though there are studies revealing that RA induces apoptosis, our experiments, in which RA alone was studied, induced only necrosis in C6 glioblastoma cells. What is more, relatively high concentrations of RA are needed to reduce their viability.

There are many studies showing that RA has antioxidant properties attenuating oxidative stress and neuronal cell death [27, 28]. Ghaffari with coworkers show that H_2_O_2_-induced cytotoxicity in N2A cells was suppressed by treatment with RA. Moreover, RA is very effective in attenuating the disruption of lactate dehydrogenase, mitochondrial membrane potential, and intracellular ROS [29]. The results of our experiments show that the amount of RS in C6 cells depends on the concentration of RA and smaller studied concentrations (50–150 *μ*M) reduced the amount of intracellular radicals. Therefore, these results confirm that, depending on the concentration, RA has an antioxidant effect and reduced the amount of intracellular radicals; thus, it can be used as a neuroprotective agent against inflammatory, neurodegenerative diseases (Alzheimer's disease and Parkinson's disease) and other biologic processes associated with enhanced ROS level. Higher examined RA concentrations (200–400 *μ*M) increased the amount of intracellular radicals in C6 cells. It is established that polyphenolic compounds often initiate cell death mechanisms by increasing the amount of ROS. Since, by studying the effect of RA on the viability of C6 cells, we have found that after the 24-hour incubation it statistically significantly reduces in the presence of 200 *μ*M concentration and higher; it could be stated that one of the mechanisms by which RA causes the death of glioblastoma C6 cells depends on ROS-sensitive pathways.

Extracts, prepared from lemon balm, not only contain rosmarinic acid, but also are enriched in other phenolic compounds. Biologically active substances identified in our extracts are protocatechuic, caftaric, caffeic, ferulic, and cichoric acids and flavonoid luteolin-7-glucoside. These our data confirm results of other investigators which found that extracts of* Melissa officinalis* are rich in phenolic compounds [30]. Naturally occurring flavonoids and phenolic acids are the hydrophilic and lipophilic nature. Investigations of chemical composition of our prepared extracts showed that the amount of identified compounds increases in the order N3 (solvent 70% ethanol) > N2 (solvent 40% ethanol) > N1 (aqueous extract).

The extract prepared in aqueous method contains amount of RA bigger than in extract N2, but amount of other biologically active substances is 2–10-fold lower than in ethanolic extracts N2 and N3. Chemical composition determined biological action: N1 reduced the viability of C6 cells significantly weaker than ethanol extracts, but ~30% stronger than RA alone. Upon studying the cell proliferation, it was found that the amount of cells after 24 hours, compared to control, does not decrease, but after 48 and 72 hours it decreases significantly. It is important to note that this extract initiates the death of cells mainly through apoptosis. The studies of intracellular RS showed that this extract, depending on the concentration, increased the amount of RS; however this increase is significantly lower, compared to the increase of RS caused by ethanol extracts. Hence, it could be true that the death of cells is initiated not only by the increased amount of intracellular RS, but also by other mechanisms.

Ethanolic (40% and 70%) extracts initiated the death of cells 4-5 times stronger than that of RA alone or aqueous extract N1. Investigations of intracellular RS concentrations demonstrated that N2 and N3 extracts have a prooxidative effect; that is, the cells treated with these extracts contained significantly more RS, compared to control (Table 2). The beneficial health effects of medicinal plants rich in polyphenols are often attributed to their potent antioxidant activities. However, it is known that medicinal plants may also exert prooxidant effects [31]. Prooxidant effects are not necessarily bad because RS may act as signalling molecules of intracellular pathways. It is known that mild prooxidant effect promotes cell proliferation and higher amounts of RS induce cell death through apoptosis or necrosis, depending on additional conditions. Our investigations with ethanolic extracts revealed that lowest concentrations of extracts investigated in our experiments (10 *μ*M RA) increased concentration of intracellular RS and proliferation of C6 cells. In cases of bigger amount of investigated extracts (40–75 *μ*M RA) higher concentrations of RS were detected, and cells dying through apoptosis and necrosis were found. Weidner with coworkers have also found that ethanolic lemon balm extract inhibits the proliferation and induces apoptosis in HT-29 and T84 human colon carcinoma cell through formation of ROS [16]. Our results as well as other authors results demonstrate that ethanolic extracts exert strong* in vitro* antitumor activity.

Taking everything into account, RA and other polyphenols identified in extracts are known as (1) radical scavengers and they could directly neutralize ROS; (2) they could also regulate intracellular antioxidant system capacity. Kim with coauthors demonstrated that RA reversed the downregulations of GSH, SOD, and Bcl-2 [32]. Therefore, it would be useful to assess and compare the effects of investigated preparations on activity of intracellular antioxidant systems.

Higher concentrations of RA and extracts (investigated concentrations) increase intracellular RS levels. Although results of our and other investigators show that RS play a significant role in cell death, it remains unclear which RS (e.g., hydrogen peroxide, superoxide, or others) are responsible for the cytotoxicity of investigated preparations. Identification of RS could be useful to hypothesise about mechanisms of action.

It is proven that RS that are generated intracellularly can induce mitochondrial depolarization and release of cytochrome c into the cytosol and thus participate in the activation of the caspase-3 cascade. So, one of regulators of cell's life/death in apoptosis and necrosis are mitochondria [33]. Several literature sources revealed that RA induced apoptosis through mitochondrial pathway [25]. Other biologically active compounds presented in extracts also may modulate mitochondrial functions, and total effect of extracts on mitochondria depends on the characteristics of complex of biologically active compounds [34, 35]. Therefore evaluation of effect of investigated preparations on mitochondrial functions could be beneficial to reveal detailed mechanisms of action in the future.

## 5. Conclusions 

RA and aqueous and ethanol extracts have a different effect on C6 cells. The cytotoxic effect of rosmarinic acid on rat glioblastoma C6 cells (LC_50_ after the incubation of 24 h 290.5 *μ*M and after 48 h LC_50_ 171.3 *μ*M) is significantly weaker, compared to differently prepared extracts from* Melissa officinalis*. RA (80–130 *μ*M) suppresses the cell proliferation and has an antioxidant effect until 150 *μ*M. 200 *μ*M and higher concentrations have a prooxidant effect and initiate the death of cells through necrosis.

The aqueous extract of lemon balm apart RA contains other biologically active substances soluble in water; however their concentration is lower if compared with ethanolic extracts. Investigated concentrations of this extract (50 *μ*M–200 *μ*M RA) have no antioxidant activity. Significantly lower concentration of aqueous extract is needed to achieve a cytotoxic activity if compare with pure RA. The cell death through apoptosis is mainly initiated.

Extracts prepared with 70% ethanol contain the biggest amount of active compounds; their concentration is lower in extract, prepared with 40% ethanol. Despite different concentrations of biologically active compounds, they have a high cytotoxic activity on glioblastoma C6 cells. These extracts initiate the generation of intracellular RS and cell death through apoptosis and necrosis.

## Figures and Tables

**Table 1:** Quantity of identified active compounds in differently prepared lemon balm extracts.

Extract type	Active compounds ± SE (*µ*g/mL)						
	Protocatechuic acid	Caftaric acid	Caffeic acid	Luteolin-7-glucoside	Ferulic acid	Cichoric acid	Rosmarinic acid
Aqueous (N1)	1.1 ± 0.12	14.2 ± 0.23	3.0 ± 0.17	1.0 ± 0.10	5.1 ± 0.21	7.9 ± 0.16	244.7 ± 1.8
Ethanolic 40% (N2)	9.84 ± 0.11	24.35 ± 1.0	39.22 ± 0.13	5.06 ± 0.11	14.73 ± 0.14	10.04 ± 0.10	152.96 ± 0.2
Ethanolic 70% (N3)	16.87 ± 0.25	67.8 ± 0.18	73.27 ± 0.16	30.40 ± 0.22	69.3 ± 0.15	49.43 ± 0.17	1750.2 ± 2.9

**Table 2:** Amount of intracellular RS increased by different concentrations of ethanolic extracts N2 and N3, %.

	Fluorescence intensity, %			
	0.5 h	1 h	1.5 h	2 h
N2				
10 *μ*M RA	15.6 ± 2.6	63.1 ± 5.2	102.9 ± 8.0	137.2 ± 11.4
40 *μ*M RA	24.4 ± 3.9	82.9 ± 7.1	138.3 ± 14.2	160.4 ± 17.2
75 *μ*M RA	35.1 ± 4.7	114.6 ± 10.5	152.4 ± 14.7	186.3 ± 15.1
N3				
10 *μ*M RA	25.5 ± 2.8	79.2 ± 6.8	117.8 ± 9.5	152.5 ± 14.6
40 *μ*M RA	34.3 ± 4.5	112.4 ± 10.3	164.7 ± 15.3	195.0 ± 18.3
75 *μ*M RA	43.8 ± 6.0	165.5 ± 13.2	208.2 ± 19.1	223.9 ± 24.7

Data presented in the table show increase of fluorescence intensity (%) depending on concentration and time. *n* = 3-4.
